# Association between sedentary behavior and Brain-Derived Neurotrophic Factor (BDNF) in children and adolescents: A protocol for systematic review and meta-analysis

**DOI:** 10.1371/journal.pone.0299024

**Published:** 2024-03-05

**Authors:** Victor Hugo de Oliveira Segundo, Kesley Pablo Morais de Azevedo, Gidyenne Christine Bandeira Silva de Medeiros, Ádala Nayana de Sousa Mata, Grasiela Piuvezam

**Affiliations:** 1 Department of Public Health, Postgraduate Program in Public Health, Federal University of Rio Grande do Norte, Natal/RN, Brazil; 2 Systematic Review and Meta-analysis Laboratory (Lab-Sys/CNPq), Federal University of Rio Grande do Norte, Natal/RN, Brazil; Universiti Malaya Fakulti Perubatan: University of Malaya Faculty of Medicine, MALAYSIA

## Abstract

**Background:**

There is evidence in the literature suggesting that high time spent in sedentary behavior (SB) can reduce the Brain-Derived Neurotrophic Factor (BDNF) levels, an important neurotrophin that plays a role in modulating cognition, learning and memory. Children and adolescents usually spend many hours a day on SB, either sitting for a long time and/or using screen equipment.

**Objective:**

The aim of this study is to describe a systematic review and meta-analysis protocol on the associations between SB and BDNF levels in children and adolescents.

**Methods:**

This protocol is guided by the Preferred Reporting Items for Systematic Reviews and Meta-Analyses Protocols and was published in the International Prospective Register of Systematic Reviews database (PROSPERO: CRD42023392246). The databases that will be searched are EMBASE, Scopus, ScienceDirect, Web of Science, SPORTDiscus, CINAHL, and PubMed. Cross-sectional and cohort studies conducted with children and adolescents (5 to 19 yr) that analyzed the association between SB and BDNF will be included in the systematic review. The characteristics of the studies, methodological aspects, and main results will be described. Then, the risk of bias (assessed by STROBE and Newcastle-Ottawa scales) and the level of evidence (assessed by the GRADE tool) from included studies will be evaluated. Sub-group analysis will also be performed. Two experienced reviewers will perform the studies selection, data extraction, and methodological quality assessment.

**Conclusion:**

This systematic review and meta-analysis will analyze the association between SB and BDNF in children and adolescents. The results will provide subsidies to better understand this relationship and will strengthen evidence-based practice for both health professionals and future researches.

## Introduction

The biological development that occurs with children and adolescents is marked by significant changes that influence different body systems [1]. In the central nervous system, this development plays a role in the way in which knowledge, skills, learning and other cognitive aspects will be incorporated into them [2,3]. However, this phenomenon is also influenced by lifestyle [4].

It is important to highlight that children and adolescents are more likely to be influenced by environmental factors (i.e. friends, school, family, community, media) in decision-making and, mainly due to the modern lifestyle, the adoption of risky behaviors has been common, which can negatively influence cognitive development [4,5].

The greater susceptibility to adopt risky behaviors as lifestyle in the first years of life are based on major endocrine changes and in brain development, especially on the immaturity of the prefrontal cortex, responsible for commanding cognitive functions [5,6]. When negative behaviors are experienced, such as sedentary behavior (SB), there may be an alteration in the Brain-Derived Neurotrophic Factor (BDNF) [7], a member of the neurotrophin protein family, which plays a role in modulating cognition, neuroplasticity, angiogenesis, and strengthening neural connectivity [8,9], increasing the chances of continuing to experience risky behaviors.

The SB can be measured by objectively (e.g., accelerometers) and subjectively (e.g., questionnaires) instruments. Objectively measured SB is defined as any waking behavior characterized by an energy expenditure of ≤ 1.5 metabolic equivalents during a sitting, reclining or lying posture. Subjectively measured SB is defined as high exposure to screen equipment while being stationary in any context and/or prolonged sitting time, information obtained through questionnaires in hours per day [10]. More than 2 hours per day of screen time (especially TV viewing) is commonly associated with adverse health and has been considered a cutoff point by health organizations [11]. In the school and domestic environment, on school transport and even during leisure time, when watching TV, using the computer or video games, talking with friends and using the cell phone, the activities are usually performed in a sitting position, with low energy expenditure [10,11].

Few studies have evaluated the association between SB and BDNF levels in young populations. While in some studies no significant association was found for healthy children [12], healthy adolescents [13] and obese children [14], in a more recent study, the authors found an association between high exposure to screen time and lower BDNF levels in obese adolescents, pointing out that this association seems to depend on factors such as the SB assessment method (objective measures or questionnaires), the type of screen equipment (TV, computer, video game, others), BDNF assessment method (serum or plasma) and body composition [7].

Previous reviews have analyzed the relationship between exercise and BDNF levels, both in cross-sectional studies [15] and in intervention studies [9,16], showing increased BDNF levels in children and adolescents that practice regular physical activity. With regard to SB, a systematic review concluded that there is conflicting evidence of the relationship between sedentary behavior and different memoryrelated outcomes among children and adults [17]. In this review, the only included study evaluating BDNF did not demonstrate any association with sedentary behavior in children [12].

A recent systematic review analyzed the association between SB and executive function in children and adolescents, and found heterogeneous results, but the authors verified that screen-based sedentary behavior tends to be negatively associated with executive function [18]. Although executive function is also mediated by BDNF levels, independent and specific analysis of the association between SB and BDNF is needed to understand other aspects of this relationship. Considering the role of BDNF in cognition and its importance in brain development, reviewing the literature on this topic is relevant.

Therefore, seeking to compile information on the associations of SB and BDNF levels in children and adolescents, the purpose of this study is to describe a systematic review and meta-analysis protocol on the subject. Depending on the characteristics of the studies that will be included in the review, subgroup analyzes will be performed seeking to understand the possible differences in the association according to the methods of evaluation of SB and BDNF, sex, body composition, types of SB and between studies with different risk of bias ratings.

## Methods

### Protocol and registration

This systematic review protocol has been registered on the PROSPERO database under the number CRD42023392246, and was prepared in accordance with the guidelines described by the Preferred Reporting Items for Systematic Reviews and Meta-Analyses Protocols (PRISMA-P) [19]. As this is a literature-based study, ethical approval is unnecessary.

### Eligibility criteria

#### Inclusion criteria

For this review, peer-reviewed journal articles that meet the eligibility criteria based on the study Population, Exposure, Comparison, and Outcome (PECO) will be considered.

The eligibility criteria for inclusion are as follows: (Population) children and adolescents aged between 5 and 19 years, of both sexes; (Exposure) objectively or subjectively measured sedentary behavior; (Comparison) not applicable; and (Outcome) BDNF levels in peripheral blood. Cross-sectional and cohort studies will be considered for inclusion.

For subjectively measured sedentary behavior will be considered self/parent-reported sitting and screen time obtained by any questionnaires. Data from wearable devices (e.g., accelerometers, cell phone) will be considered for objectively measures of sedentary behavior. With regard to BDNF, serum and plasma levels will be considered.

The World Health Organization defines children as those aged 0 to 9 years and adolescents as those aged 10 to 19 years. However, for the present review protocol, we defined that only studies with children aged between 5 to 9 years will be included, considering that from this age (5 years old) onwards they have a better established routine, including going to school, eating patterns and physical activities, important aspects for the variables that will be studied in the review [20].

#### Exclusion criteria

Studies performed with children and adolescents who have physical or intellectual disabilities or with psychological or neuroendocrine problems will be excluded. Studies that do not present sufficient data regarding the sample characteristics, data collection and analysis procedures will also be excluded if the information is not obtainable via e-mail with the authors. Attention will also be given to articles that may be the result of the same study and, if confirmed, one of them will be excluded.

### Information sources and literature search

Initially, exploratory searches will be carried out in the databases using different terms. To define the search equation, different keywords indexed in the Medical Subject Headings (MeSH) and other synonymous terms related to population, exposure and outcome will be used (Table 1).

**Table 1:** Preliminary pilot search strategy in MEDLINE/PubMed.

Description	Terms
#1 Population	(Child OR Adolescent OR Teenagers OR Youth)
#2 Exposure	(“Sedentary behavior” OR “Sedentary time” OR “Sedentary lifestyle” OR “Screen time” OR Sitting OR Television OR TV OR “Computer time” OR Media OR Physical inactivity)
#3 Outcome	(“Brain-derived neurotrophic factor” OR BDNF OR Neurotroph* OR “Neuronal activity” OR Platelets OR Serum)
Search equation	#1 AND #2 AND #3

The searches will be conducted in the following electronic bibliographic databases: EMBASE, Scopus, ScienceDirect, Web of Science, SPORTDiscus, CINAHL, and PubMed. The search equation will be created based on the keywords and the combination of OR and AND Boolean operators, according to the characteristics of each database. In each database, the title, abstract, and keywords search fields will be searched. The searches will be limited to the English language and with no year restriction.

Two reviewers will independently select all literatures according to the predesigned eligibility criteria. After searches, the EndNote^®^ bibliographical reference manager (version X9, Clarivate Analytics) will be used to compile the articles and duplicates will be removed. Titles and abstracts of identified articles will be checked for relevance in the first and second stages of screening, respectively. In the third stage, full-text articles will be retrieved and considered for inclusion. Additionally, references cited in articles will be reviewed to locate any additional relevant articles not retrieved within the primary search.

**Fig 1:**
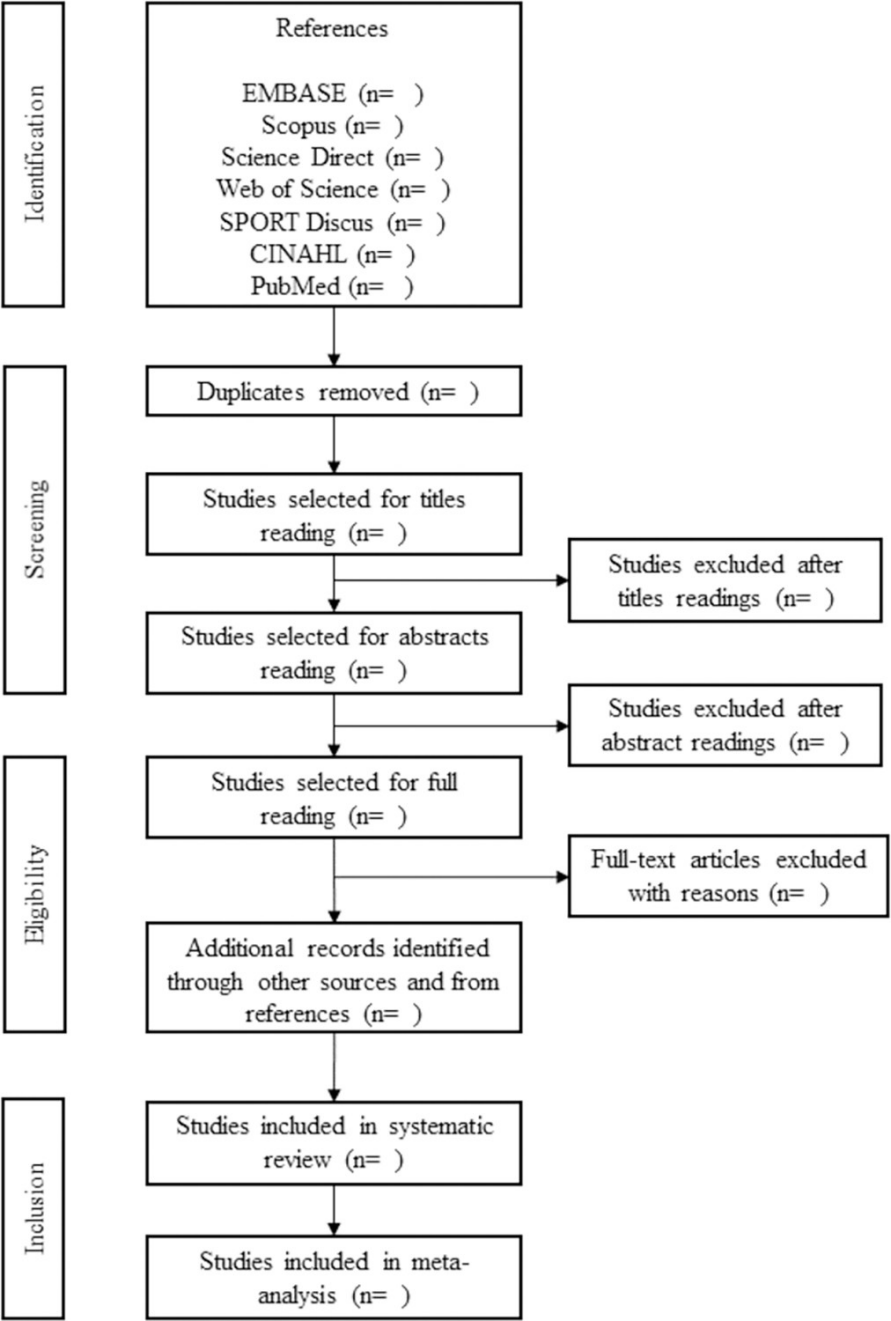
Article selection flowchart. Adapted from PRISMA-P [19].

Two researchers will carry out all steps, independently. Any divergences between two reviewers will be settled down by discussion with a third reviewer. All information on the phases of the selection process are identified in Fig 1.

### Data extraction

Information on the reference (country, authors, and year of publication); sample characteristics (number of participants, sex, and age); methodological aspects as study design, assessment method of sedentary behavior (device or questionnaire used), type of sedentary behavior (e.g., TV viewing, using a computer, playing video games, sitting time at school or at home), assessment method of BDNF (serum or plasma), and adjusted variables; and main results (statistical results and main conclusions) of the selected studies will be collected by two reviewers who will fill in a previously defined form in an EXCEL spreadsheet.

Regarding exposure and outcome measures, for SB, both continuous measures (e.g., minutes per day) and categorical measures (e.g., more than 2 hours of exposure to screens per day will be categorized as high SB) will be considered. For serum and plasma BDNF, studies that included continuous measurements in both nanograms per milliliter (ng/mL) and picograms per milliliter (pg/mL) of blood will be considered.

All doubts about specific points or differences in results can be clarified with the help of the third researcher. In cases of missing data, the corresponding author will be contacted three times by email, which will be sent at intervals of 2 weeks.

### Risk of bias, methodological quality, and Grading of Recommendations Assessment, Development, and Evaluation assessments

The risk of bias of included studies will be analysed by two reviewers. The risk of bias will be assessed through critical analysis of the domains related to sample selection, study context, outcomes, statistical analysis and results included in the methodological quality assessment instruments.

To assess the methodological quality of the cross-sectional and cohort studies, will be used the Strengthening the Reporting of Observational Studies in Epidemiology (STROBE) [21] and the Newcastle-Ottawa (NOS) [22] scales, respectively.

The STROBE checklist [21] contains 22 items that comprise different sections of a manuscript: title, abstract, introduction, methods, result and discussion. The methodological quality will be classified as (a) high: when the article presents sufficient data on the evaluated criteria; (b) moderate: when the study presents incomplete data on the evaluated items); and (c) low: when the study does not provide enough data to understand the items.

The NOS scale [22] evaluates three quality parameters (selection, comparability, and outcome) divided in eight specific items. Each item on the scale scores 1 point, except for comparability, which can be scored up to 2 points, and the total score for each article can reach 9 points. For this review, articles scoring 7 to 9 will be considered to be of high methodological quality; moderate quality articles scoring 5 to 6; and low methodological quality articles scoring below 4 points.

No study will be excluded according to its methodological quality or risk of bias, but these aspects will be considered in the discussion of the review. Depending on the number of included studies and risk of bias ranking, a subgroup analysis between studies with different risk of bias may be considered.

The level of evidence assessment and strength of the studies recommendations will be performed using the Grading of Recommendations Assessment, Development, and Evaluation (GRADE) tool, allowing each included article to be classified as very low, low, moderate, and high quality of evidence [23].

All analyses will be carried out by two reviewers, with the help of a third researcher in case of discrepancy. The reviewers will be previously trained and calibrated to ensure uniformity in the evaluation of the criteria, and a kappa index will be applied for agreement analysis.

### Data analysis and synthesis

Data will be summarized through a narrative approach, and the characteristics of the included studies will be described in tables.

To perform the meta-analysis, a minimum of two studies will be considered [24], and Review Manager^®^ (version 5.3, Cochrane Collaboration) will be used. Separate meta-analyses will be performed for cross-sectional and cohort studies. If cohort studies have multiple assessment time points, the first and last assessment will be considered. A significance level of p<0.05 will be considered for all tests that will be performed in this meta-analysis.

The evaluation of heterogeneity between studies will be verified by statistical tests χ^2^ and I^2^. The I^2^ evaluates the proportion of variability between studies and values of <25%, 50% and >75% will be considered as low, moderate and high inconsistency, respectively [25]. If high heterogeneity is verified, then a narrative synthesis will be undertaken.

Meta-regression analyses will be performed to assess the effect of study quality (STROBE and NOS scales) on the calculated estimates. Sensitivity analyses will be performed by excluding each study at a time in order to assess the influence on the pooled effect size. Funnel plots will be used to assess publication bias [26]. A linear regression approach will be used to evaluate funnel plot asymmetry [27]. To perform the meta-regression and the publication bias assessment, we will consider a minimum of 10 included studies. The random effects model will be applied using the method of DerSimonian and Laird [28] to calculate the total effect size of the studies included in the meta-analysis.

Moreover, depending on the quantity and heterogeneity of the included studies, subgroup analysis will be performed for age (children aged 5–9 years and adolescents aged 10–19 years), sex (boys and girls), nutritional status (e.g. normal weight, overweight and obese), by the way in which the SB (e.g., objective or self-reported) and BDNF (e.g. serum or plasma) levels are evaluated, by the type of SB (e.g., TV viewing, playing video games, using computer), and also between studies with different risk of bias.

## Discussion

The proposed systematic review and meta-analysis will present the results of studies that evaluated the association between SB and BDNF levels in children and adolescents. Recent studies have raised methodological hypotheses that can influence BDNF levels [7,17], and the analysis of these variables can support better decision-making for future studies or interventions aimed at this population.

BDNF plays a crucial role in the development, maintenance and plasticity of the central and peripheral nervous system [8], and has been used to investigate the mechanisms that act in the regulation of decision-making in younger populations [7,9]. Low levels of BDNF can affect cognitive health and have been associated with cognitive impairment [29,30], difficulties in learning and memory processes [31] and greater chances of making unhealthy decisions [32]. When the ability to control decision-making is reduced, the chances of choosing to follow risky behaviors increase, as the pleasurable sensations of reward become more evident.

For children and adolescents, who are in full brain development and going through a great period of neuroplasticity, the tendency to maintain sedentary behaviors such as excessive sitting time and excessive time using screen equipment also increases [7,33]. Furthermore, SB during childhood and adolescence is one of the risk factors for triggering cardiovascular diseases in adult life, which makes it relevant to develop strategies to control and reduce SB since the first years of life [34,35].

Cross-sectional studies that assessed objective measures of SB found null associations with BDNF in obese children [14] and healthy children [12]. A study with adolescents found association between high rates of SB with lower serum BDNF levels in boys, but not in girls [13]. In the studies that assessed screen based SB, no association was observed between TV viewing and BDNF in healthy children [12], but in a study with adolescents, higher exposure to TV viewing was associated with lower serum BDNF levels, while it was not observed in other screen equipment [7]. These findings demonstrate the variability that exists in the studies, which a systematic review could better elucidate this relationship and contribute to decision-making in new studies.

It is important to highlight that other systematic review studies identified higher levels of BDNF in children and adolescents who practice physical activity [9,15]. Although a few studies included in these reviews have not observed differences, especially for strength exercises, in general, the regular practice of physical activity has been shown to increase peripheral BDNF levels. Additionally, a recent meta-analysis found that high-intensity interval training (HIIT) was more effective in increasing BDNF concentrations in healthy adults, indicating that intensity is an important variable in this scenario [36].

Our hypothesis is based on this premise, considering that high levels of sedentary behavior may present lower BDNF levels. Also, based on observations from other authors, there is a hypothesis that the type of SB may influence BDNF levels, where possibly "active" sedentary behaviors (those in which the subject is in SB but performs some cognitive task simultaneously, such as playing video games, using the computer to study, and others) [7,37,38]. In this perspective, this protocol intends to overcome this gap, deepening through subgroup analysis taking into account the variability of the measuring instruments, types of SB and the characteristics of the participants.

Systematic review studies may have some limitations, which involve biases in the procedures for searching, registering, extracting and analyzing data. There is also risk of bias in the presentation of results, often due to the quality of the included studies and even due to the possible inexperience of the reviewers. However, to avoid biases in conducting this review, all PRISMA guidelines will be followed, respecting the completion of the steps in pairs, always with a third reviewer to discuss possible divergences. The checklists and softwares that will be used to carry out methodological quality analyses and statistical analyses are also validated and recognized by the scientific community. Finally, the team of researchers involved has notable experience in conducting systematic review and meta-analysis studies, which can be considered one of the strengths of this study.

## Conclusion

The protocol for this systematic review and meta-analysis is presented in a clear and systematic way for extracting information and presenting relevant results on the association of SB and BDNF levels in children and adolescents. With the intention of concluding and quickly publishing the systematic review resulting from this protocol, we infer that the results will support further research with more structured and summarized information.

## Supporting information

S1 Checklist PRISMA-P 2015 checklist. (DOCX)
